# Characteristics associated with low risk of significant coronary stenosis after an episode of acute coronary syndrome

**DOI:** 10.1186/2197-425X-3-S1-A754

**Published:** 2015-10-01

**Authors:** R Blancas, Ó Martínez González, M Chana, D Ballesteros, B López Matamal, MÁ Alonso, C Martín Parra

**Affiliations:** Critical Care, Hospital Universitario del Tajo, Aranjuez, Spain

## Introduction

Over 15% of patients admitted for acute coronary syndrome have absent or non-significant coronary lesions [1]. Anyway, intensive antithrombotic treatment and invasive procedures are planned in these patients. It would be important to have some elements to predict patients at low risk for coronary stenosis.

## Objectives

To describe the characteristics and outcome of patients admitted for acute coronary syndrome (ACS) with absent or non-significant coronary lesions.

## Methods

Patients admitted to the ICCU for ACS between 2008 and November 2014 were included. We compared the group with absent or non-significant coronary lesions (< 50% coronary lumen stenosis) with the group with significant lesions. Variables were compared using the Chi-square test or Fisher´s exact test where appropriate. P value was considered statistically significant when p < 0.05.

## Results

Three hundred and twenty-five patients were admitted for ACS. Those patients to whom coronary angiography was not performed (39 patients) and those admissions following the first one of each patient were excluded from the statistical analysis. The mean age was 62.96 ± 13.18 years

(64.8 ± 12.6 for group with normal coronary arteries or non-significant lesions, 64.99 ± 13.32 for the group with significant injuries, p = 0.217). Females represented 28% of patients. Coronary catheterization showed no lesions in 37 patients (12.9%) and non-significant lesions in 1 (3.8%). The variables associated with the presence of normal coronary arteries or non-significant lesions were: female gender, pretreatment with dicoumarol, digoxin or nitrates, non-inferior-posterior presence of ischemia on EKG and negative markers of myocardial necrosis. Both groups had a similar outcome in ICCU except for the developement of new myocardial infarction, which could be related to percutaneous revascularization.

## Conclusions

Some conditions in patients admitted for ACS may warn of the possibility of the absence of significant lesions through the coronary tree. While it is necessary to consider alternative diagnoses in these patients, their outcome in UCCI does not differ from those susceptible to coronary revascularization.
